# Regional and seasonal partitioning of water and temperature controls on global land carbon uptake variability

**DOI:** 10.1038/s41467-022-31175-w

**Published:** 2022-06-16

**Authors:** Kai Wang, Ana Bastos, Philippe Ciais, Xuhui Wang, Christian Rödenbeck, Pierre Gentine, Frédéric Chevallier, Vincent W. Humphrey, Chris Huntingford, Michael O’Sullivan, Sonia I. Seneviratne, Stephen Sitch, Shilong Piao

**Affiliations:** 1 Sino-French Institute for Earth System Science, College of Urban and Environmental Sciences, Peking University, Beijing, 100871 China; 2 Department of Biogeochemical Integration, Max Planck Institute for Biogeochemistry, Jena, 07745 Germany; 3 Laboratoire des Sciences du Climat et de l’Environnement, LSCE/IPSL, CEA-CNRS-UVSQ, Université Paris-Saclay, Gif-sur-Yvette, 91191 France; 4 The Cyprus Institute, 20 Konstantinou Kavafi Street, Nicosia, 2121 Cyprus; 5 Department of Biogeochemical Systems, Max Planck Institute for Biogeochemistry, Jena, 07745 Germany; 6 Department of Earth and Environmental Engineering, Columbia University, New York, NY 10027 USA; 7 Division of Geological and Planetary Sciences, California Institute of Technology, Pasadena, CA 91125 USA; 8 U.K. Centre for Ecology and Hydrology, Benson Lane, Wallingford, OX10 8BB UK; 9 College of Engineering, Mathematics and Physical Sciences, University of Exeter, Exeter, EX4 4QF UK; 10 Department of Environmental Systems Science, Institute for Atmospheric and Climate Science, ETH Zurich, Zurich, 8092 Switzerland; 11 College of Life and Environmental Sciences, University of Exeter, Exeter, EX4 4RJ UK; 12 State Key Laboratory of Tibetan Plateau Earth System and Resources Environment (TPESRE), Institute of Tibetan Plateau Research, Chinese Academy of Sciences, Beijing, 100085 China

**Keywords:** Carbon cycle, Carbon cycle

## Abstract

Global fluctuations in annual land carbon uptake (NEE_IAV_) depend on water and temperature variability, yet debate remains about local and seasonal controls of the global dependences. Here, we quantify regional and seasonal contributions to the correlations of globally-averaged NEE_IAV_ against terrestrial water storage (TWS) and temperature, and respective uncertainties, using three approaches: atmospheric inversions, process-based vegetation models, and data-driven models. The three approaches agree that the tropics contribute over 63% of the global correlations, but differ on the dominant driver of the global NEE_IAV_, because they disagree on seasonal temperature effects in the Northern Hemisphere (NH, >25°N). In the NH, inversions and process-based models show inter-seasonal compensation of temperature effects, inducing a global TWS dominance supported by observations. Data-driven models show weaker seasonal compensation, thereby estimating a global temperature dominance. We provide a roadmap to fully understand drivers of global NEE_IAV_ and discuss their implications for future carbon–climate feedbacks.

## Abstract (web summary)

The dominant driver of variations in global land carbon sink remains unclear. Here the authors show that the seasonal compensation of temperature effects on land carbon sink in the Northern Hemisphere could induce a global water dominance.

## Introduction

The global land carbon sink offsets about 30% of anthropogenic CO_2_ emissions every year, slowing global warming^1,2^. However, this sink exhibits substantial interannual variability (IAV), which causes the CO_2_ growth rate (CGR) to fluctuate^3,4^. Understanding the IAV of the net land carbon sink, hereafter the IAV of net ecosystem exchange (NEE_IAV_), is critical for estimating the future evolution of atmospheric CO_2_ with the emergence of carbon–climate feedbacks^5–7^. Such an understanding is especially useful if climate-driven fluctuations of land carbon sink become more frequent or more intense under climate change, which may impact the capability of the land to offset CO_2_ emissions. The dominant drivers of the global NEE_IAV_ appear to be inconsistent across studies^8–10^. Some studies have shown that the global NEE_IAV_ represented by the IAV of the CGR, is strongly related to the variations of the mean annual temperature^11,12^. Other studies underlined the dominance of terrestrial water storage (TWS) and thus water availability for vegetation, even when the temperature (T) was accounted for as a co-predictor^9,13^.

This apparent controversy among research findings arises from different assessments of ecosystem responses to water availability or T in different regions and seasons, which combine in complex ways to produce the emergence of global drivers of NEE_IAV_^10,14–16^. Hence depending on the datasets used, their aggregation can lead to differing conclusions about the dominant drivers for the global NEE_IAV_. How the distinct contributions of each region sum to the global signal has not been investigated in detail. Most studies have focused on the response of tropical net ecosystem exchange (NEE) to T and water availability^4,12,17–19^. Researchers have placed far less attention on the contribution of northern ecosystems to the global response, despite evidence identifying large NEE_IAV_ over North America and Europe in response to interannual variability of both T and water availability^20–23^. Furthermore, a full understanding of seasonally varying influences of water availability and T on NEE_IAV_ for tropical and northern ecosystems is still lacking^15,21,24^. Water availability has been suggested to be positively correlated with carbon sink anomalies in most seasons over North America, while seasonally opposite (i.e., compensating) influences of T on NEE may weaken the overall impact of T on annual NEE^20^. Seasonal compensation effects of T on NEE were reported during recent extreme events in northern regions^20,25,26^. For example, higher net carbon uptake induced by the warmer T in spring was found to be canceled out by less carbon uptake in the following warmer summer, given a legacy drying of soils from spring to summer^25,27^. It remains unclear to what extent such a seasonal compensation effect of T on NEE influences the overall global response of NEE_IAV_ to annual T anomalies.

Here we assessed how the impacts of TWS and T on the NEE_IAV_ vary in different regions and seasons, and how these impacts aggregate in space and time to influence the global annual relationship of NEE with TWS and T. To do so, we used three spatially and monthly explicit estimates of NEE. These datasets are (1) three atmospheric inversions (CAMS, Jena CarboScope, and a new Jena CarboScope inversion called NEE-T-TWS)^28–31^, (2) dynamic global vegetation models (DGVMs) of the TRENDY project version 7 (refs. ^1,32^), and (3) three machine learning models upscaling NEE from in situ eddy-covariance measurements (FLUXCOM)^10,33^. All of these datasets cover the period from 1979 to 2016. Although the NEE from atmospheric inversions includes emissions from disturbances such as fires, which are not included in FLUXCOM models^33^ and some TRENDY models^1^, fire emissions are known to have a relatively small impact on the global NEE_IAV_^4^. We focused on determining the contribution from tropical and northern ecosystems to the dependence of global NEE_IAV_ on TWS and T. For tropical ecosystems, we examined the contributions of NEE_IAV_ during the dry and the wet seasons. For northern ecosystems, we considered four seasons separately (boreal spring, summer, autumn, and winter). Throughout this paper, we used the sign convention that a positive value of NEE is for a net carbon release from land to the atmosphere.

## Results

### Annual global NEE_IAV_ correlations with TWS and T (r_TWS_ and r_T_)

**Fig. 1:**
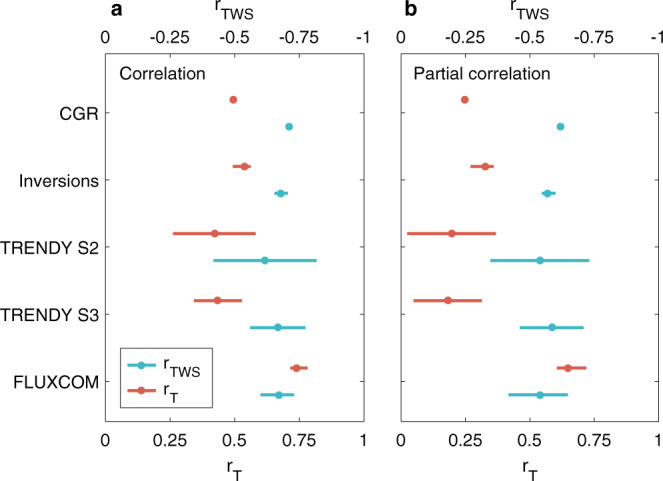
Relationships between the interannual variability (IAV) of global net ecosystem exchange (NEE) and terrestrial water storage (TWS) or temperature (T). The correlation (**a**) and partial correlation (**b**) between the IAV of global NEE and the IAVs of global mean TWS (r_TWS_) or T (r_T_) are estimated. The IAV of CO_2_ growth rate (CGR) is a proxy of the IAV of global NEE. The NEE is also estimated by atmospheric inversions, dynamic global vegetation models (DGVMs) from the TRENDY project in simulation S2 (NEE output; see Methods) and in simulation S3 (NBP output with the opposite sign; see Methods), and FLUXCOM models. Cyan (red) circles indicate the correlations between global NEE and TWS (T). Error bars for atmospheric inversions and FLUXCOM models indicate the range of models, while the error bars for DGVMs from the TRENDY project are the 1-σ inter-model spread. The NEE derived from FLUXCOM models and DGVMs from the TRENDY project in S2 (NEE output) do not include disturbances (e.g., fire emissions), but the NEE in atmospheric inversions and DGVMs from the TRENDY project in S3 (NBP output with the opposite sign) include disturbances. A larger NEE indicates less net carbon uptake.

We first calculated the correlation between the annual global NEE_IAV_ and the IAV of TWS (r_TWS_) or T (r_T_), from CGR observations (see Methods) and three different NEE datasets listed above, using reconstructed TWS based on the Gravity Recovery and Climate Experiment (GRACE) satellite observations^34^ and T from a gridded record of meteorological measurements^35^. We found that at the annual timescale, the global NEE_IAV_ was more strongly correlated with TWS than with T when using data from CGR observations, atmospheric inversions, and DGVMs from TRENDY project (Fig. 1a). This result agrees with the findings of Humphrey et al.^13^, which were based on CGR time series as a proxy of the global NEE_IAV_. The strong correlation of NEE with TWS may, however, contain some response to T because of the strong covariation between TWS and T. These covariations may occur especially over shorter timescales because drought and heatwaves usually co-occur as compound events^36^. Furthermore, atmospheric feedbacks induced by soil moisture deficits produce increases in T and atmospheric dryness that affect the global NEE_IAV_^9^. To filter out the impact of T on the relationship between NEE and TWS and vice versa, we calculated the partial correlations between the global NEE_IAV_ and the individual IAVs of T and TWS, after controlling for the effect of the other variable. These results also show that the global NEE_IAV_, as estimated by CGR observations, atmospheric inversions, and DGVMs, is more strongly linked to TWS than T (Fig. 1b).

By contrast, both simple correlation and partial correlation results show that the global r_T_ value is larger than the absolute value of global r_TWS_ when using the NEE values of FLUXCOM models (Fig. 1). The more substantial control of temperature on FLUXCOM-estimated global NEE_IAV_, which is opposite to results from atmospheric inversions and DGVMs, links to a higher NEE_IAV_ correlation with T rather than a lower correlation with TWS. Indeed, the value of global r_TWS_ obtained from FLUXCOM models (r_TWS_ = −0.67) lies in the range of the r_TWS_ values from other datasets in Fig. 1a. In addition, the difference between the simple and partial correlation values with T is generally much larger than with TWS, except for FLUXCOM models (Fig. 1a versus b). This finding suggests a noticeable contribution of TWS to the correlation between global NEE_IAV_ and T. This contribution of TWS is also supported by previous results from CGR observations and coupled land–atmosphere models^9,13^.

### Seasonal compensation effects in northern ecosystems

**Fig. 2:**
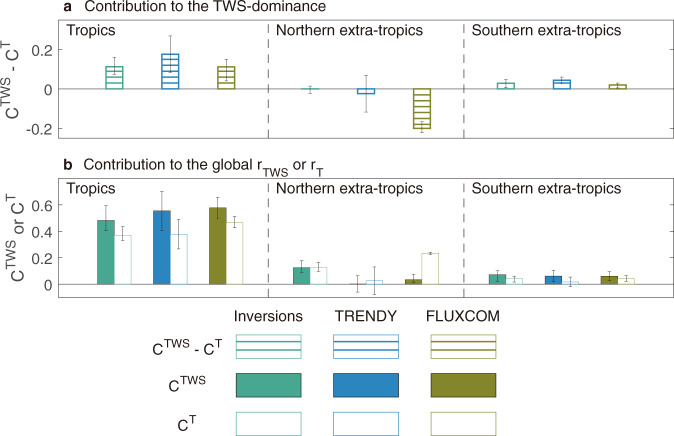
Contribution of three latitudinal bands to the relationships between global interannual variability of net ecosystem exchange (NEE_IAV_) and terrestrial water storage (r_TWS_) or temperature (r_T_). In panel **a**, positive values mean that the NEE_IAV_ in the latitudinal band supports the dominance of terrestrial water storage in the global NEE_IAV_ while the negative values mean that the NEE_IAV_ in the latitudinal band supports the dominance of temperature. In panel **b**, the solid bars are the contributions to the global r_TWS_ (C^TWS^) while open bars are the contributions to the global r_T_ (C^T^). Positive values of C^TWS^ or C^T^ mean that removing the regional NEE_IAV_ would reduce the absolute value of the global r_TWS_ or r_T_. In panels **a** and **b**, green bars indicate NEE_IAV_ is estimated by atmospheric inversions, while blue bars and brown bars indicate that NEE_IAV_ is estimated by DGVMs from the TRENDY project in simulation S2 (NEE output) and by FLUXCOM models, respectively. For atmospheric inversions and FLUXCOM models, the error bars indicate the range of models while for DGVMs from the TRENDY project, the error bars indicate the 1-σ inter-model spread.

To further investigate the relative dominance of TWS and T in the overall global NEE_IAV_, we disaggregated the annual global NEE_IAV_ correlations with TWS and T (r_TWS_ and r_T_) into contributions from different regions and seasons (named C^TWS^ and C^T^). Our disaggregation method exploits the additive properties of covariances (see Methods). Briefly, the sum of all local and seasonal C^TWS^ and C^T^ values explains the absolute value of the global r_TWS_ and r_T_, respectively. Positive values of C^TWS^ or C^T^ mean that removing NEE from a given region/season reduces the absolute value of the global r_TWS_ or r_T_. As shown in Fig. 2a, all three approaches used to diagnose regional NEE agree on a larger C^TWS^ than C^T^ in the tropics and the southern extra-tropics. Therefore, the higher global r_T_ value than the absolute value of r_TWS_ diagnosed from FLUXCOM models must be the consequence of a distinct modulation of C^TWS^ and C^T^ in the Northern Hemisphere (NH, >25°N). This leads us to examine more in-depth the contribution of NEE_IAV_ in the NH to the global r_TWS_ and r_T_. We found that FLUXCOM models did estimate a larger C^T^ for the NH (C^T^ = 0.23 ±  0.01) than atmospheric inversions (C^T^ = 0.12 ± 0.04) and DGVMs from TRENDY project in their simulation S2 (C^T^ = 0.03 ± 0.11) (Fig. 2b). In addition, the C^T^ of northern ecosystems in FLUXCOM models is also much higher than C^TWS^, while in atmospheric inversions and DGVMs, the C^T^ of northern ecosystems is almost equal to C^TWS^ (Fig. 2a). This finding clearly illustrates that the dominance of T (rather than TWS) in the global NEE_IAV_ estimated by FLUXCOM models takes its origins in the NH.

**Fig. 3:**
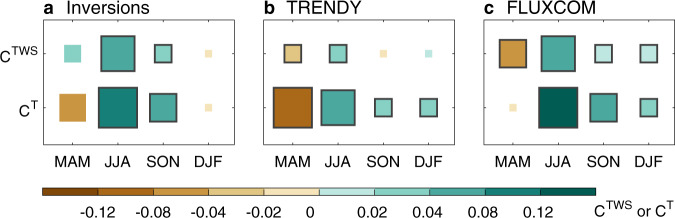
C^TWS^ and C^T^ in the northern extra-tropics during four seasons. C^TWS^ and C^T^ indicate contributions to the global correlations of interannual variability of net ecosystem exchange (NEE_IAV_) against terrestrial water storage (r_TWS_) and temperature (r_T_), respectively. NEE_IAV_ is estimated by atmospheric inversions (**a**), DGVMs from the TRENDY project in simulation S2 (NEE output) (**b**), and FLUXCOM models (**c**). The four seasons are boreal spring (MAM, March–May), summer (JJA, June–August), autumn (SON, September–November), and winter (DJF, December and January–February). Positive values of C^TWS^ or C^T^ mean that removing the seasonal NEE_IAV_ would reduce the absolute value of the global correlation r_TWS_ or r_T_. The larger rectangles indicate the higher absolute values of the C^TWS^ or C^T^. For atmospheric inversions and FLUXCOM models, the black edges of the rectangles indicate that the signs of the C^TWS^ or C^T^ are the same among all models. For DGVMs from the TRENDY project, black edges of the rectangles indicate that the signs of C^TWS^ or C^T^ derived from more than 10 out of 14 models are consistent with those from the model ensemble mean.

To get more insights into this NH phenomenon, we then assessed how the large annual C^T^ of northern ecosystems suggested by FLUXCOM models emerges from different seasons (boreal spring, summer, autumn, and winter). As shown in Fig. 3, all three approaches agree that C^T^ in summer is the largest contributor to the annual C^T^ in the NH, as it is much higher than C^T^ in autumn and winter. This implies a relatively weak role of ecosystem respiration during the non-growing season in the annual NEE_IAV_. The high C^T^ of the NH in summer is however partially compensated by C^T^ in spring from all three datasets (Fig. 3). Compared to atmospheric inversions and DGVMs from the TRENDY project in simulation S2, FLUXCOM models instead estimate a much weaker spring-summer compensation of seasonal C^T^ in northern ecosystems. Specifically, the FLUXCOM-estimated C^T^ value in summer is larger than in atmospheric inversions and DGVMs, while in spring, it is much weaker with a near-zero value (Fig. 3c). This explains the emerging large annual C^T^ in northern extra-tropical regions (Fig. 2), and thus indirectly the T-dominance in the global NEE_IAV_ in FLUXCOM models by adding to the tropical influence (Fig. 1).

**Fig. 4:**
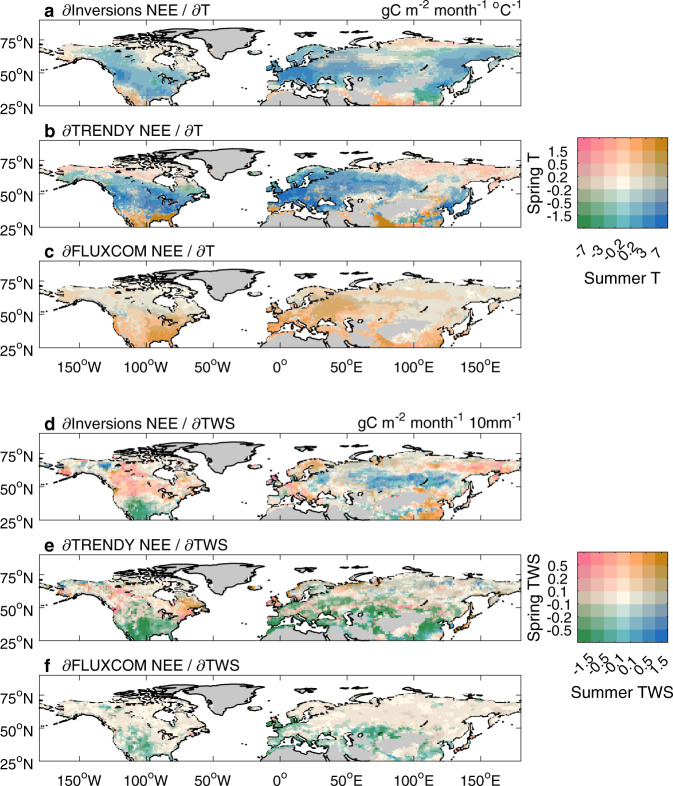
Sensitivity of net ecosystem exchange (NEE) to temperature (T) and terrestrial water storage (TWS) in boreal spring and summer. NEE is estimated by atmospheric inversions (**a**, **d**), DGVMs from the TRENDY project in simulation S2 (NEE output) (**b**, **e**), and FLUXCOM models (**c**, **f**). A positive sensitivity to TWS means drier conditions are correlated with more net carbon uptake, while a positive sensitivity to T means warmer conditions are correlated with less net carbon uptake.

We have identified that the strength of the spring-summer compensation of C^T^ in northern ecosystems is largely related to the dominant driver of the global NEE_IAV_. Therefore, we next investigated more systematically the magnitude of C^T^ in spring and summer across the NH. To do so, we examined the sensitivities of NEE to T in boreal spring and summer. Results show that in summer, all three approaches agree on a smaller net carbon uptake with warmer T anomalies at northern mid-latitudes (Fig. 4a–c; brown or blue areas), but they are not consistent at northern high latitudes (Fig. 4a–c). On the one hand, atmospheric inversions and DGVMs from TRENDY project in simulation S2 tend to suggest a negative T sensitivity (warmer summer, lower NEE, and more uptake) in the boreal and arctic ecosystems of North Asia (Fig. 4a, b; pink or green areas), which is the opposite of their results over the mid-latitudes. This estimation of negative T sensitivity by DGVMs could be influenced by their lack of permafrost carbon dynamics and nutrient limitation in their simulations^1,37,38^. Atmospheric inversions may be also limited by relatively sparse atmospheric CO_2_ measurement stations in northern Eurasia (Supplementary Note 1). Nevertheless, a negative summer T sensitivity of NEE in high-latitude ecosystems is not implausible.

On the other hand, FLUXCOM models indicate that summer T effects on NEE at high latitudes have the same sign as those in the mid-latitude regions (warmer summer, higher NEE, and less uptake; Fig. 4c; brown areas), which could explain the higher overall summer C^T^ of northern ecosystems than that estimated by atmospheric inversions and DGVMs from the TRENDY project (Figs. 3c and 4c). To what extent this is because FLUXCOM models are trained by dense flux tower data in temperate regions and extrapolate temperate NEE responses to the high-latitude regions needs to be further studied. A recent study based on eddy-covariance measurements found that higher summer temperature could enhance the net carbon uptake in the Arctic^39^, more consistent with atmospheric inversions and DGVMs rather than FLUXCOM models. Given the diversity of boreal and arctic systems^40–42^, we note that the response of NEE to summer T in the high-latitude regions still remains uncertain.

Compared with summer, the sensitivity of spring NEE to spring T anomalies estimated by FLUXCOM models is different from estimates by the other two approaches almost throughout the NH. In general, atmospheric inversions and DGVMs from the TRENDY project show a negative sensitivity of NEE to spring T (warmer spring, lower NEE, and more uptake) (Fig. 4a, b; green or blue areas), while the T sensitivity from FLUXCOM models is positive (warmer spring, higher NEE, and less uptake) (Fig. 4c; brown areas). Such a widespread positive sensitivity of NEE to spring T estimated by FLUXCOM models is surprising because a warmer spring (i.e., higher T) is commonly observed to enhance the net carbon sink at most long-term eddy-covariance sites in northern ecosystems^16,43^. This FLUXCOM-estimated positive sensitivity may be due to the temporal extrapolation of the T sensitivity from some short-term sites, where the impact of T on NEE in recent years could be different from the long-term average of the impact of T^44,45^. There may also be an effect whereby the training of FLUXCOM models by both space and time gradients of NEE, may cause subtropical sites to overly affect the prediction of the T sensitivity at temperate and boreal sites^46^. The positive sensitivity of NEE to spring T estimated by FLUXCOM models results in the spring C^T^ being positive in some regions of NH, which weakens the overall compensation effects of spring C^T^ on summer C^T^ (Fig. 3c). Hence FLUXCOM models estimate the global NEE_IAV_ to be more correlated with T instead of TWS (Fig. 1).

The results from atmospheric inversions and DGVMs from the TRENDY project demonstrate that the seasonal (spring vs. summer) compensation of the effects of T anomalies on NEE_IAV_ in northern ecosystems weakens the control of global T on global NEE_IAV_. This seasonal compensation also weakens the influence of annual T on NEE_IAV_ in the NH, thereby explaining the previous findings that the control of global T on global NEE_IAV_ is weaker than that of tropical T^4,13^. The seasonal compensation in NH systems occurs mainly between the boreal spring and summer, as indicated by atmospheric inversions and DGVMs from the TRENDY project (Fig. 3). A warmer spring can stimulate carbon uptake in the NH^16,47,48^, causing the contribution of the NH in spring to the global r_T_ to be negative. In contrast, warmer summers with potentially higher air temperature than the optimum of vegetation productivity or reduced soil moisture can suppress the net carbon uptake in northern ecosystems^49–52^. The reduced net carbon uptake (higher NEE) in warmer summers results in a positive contribution of NEE_IAV_ in northern ecosystems to the global r_T_. In addition, atmospheric inversions and DGVMs from the TRENDY project indicate that the seasonal compensation of the effects of T on NEE_IAV_ is not limited to local extreme events such as severe droughts^20,25,27^, but is also a widespread feature arising during normal years in the NH. This compensation of T effects within the NH explains why at the global scale, TWS is the strongest driver of annual variations in NEE, except in FLUXCOM models (Fig. 1). The possible underestimation of NH seasonal compensation effects in FLUXCOM models leads to a dominant role of T rather than TWS in the global NEE_IAV_ from this approach, which is in contrast to the results obtained using the observed CGR^13^ (Fig. 1).

### Contribution of tropical NEE during the dry and wet seasons

**Fig. 5:**
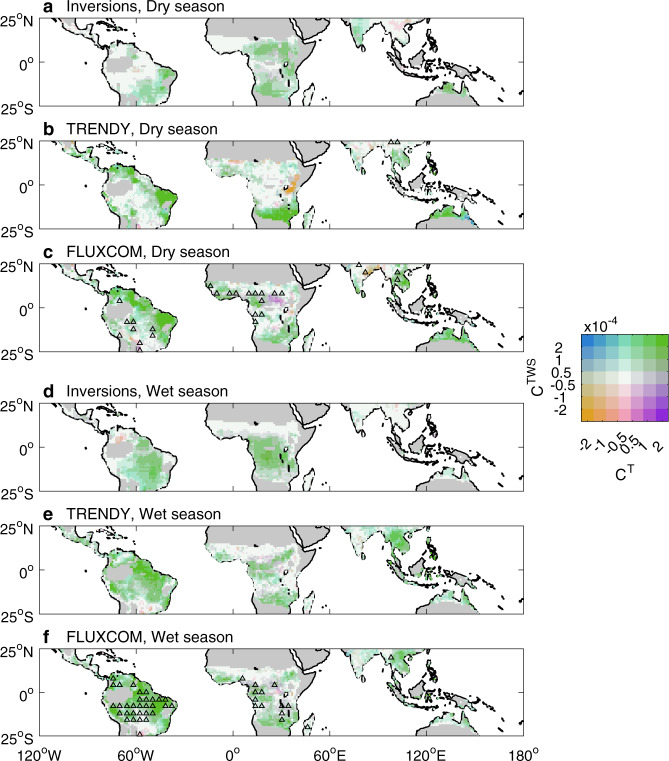
Spatial distribution of the C^TWS^ and C^T^ in the tropics during the dry and wet seasons. C^TWS^ and C^T^ indicate contributions to the global correlations of interannual variability of net ecosystem exchange (NEE_IAV_) against terrestrial water storage and temperature, respectively. NEE_IAV_ is estimated by atmospheric inversions (**a**, **d**), DGVMs from the TRENDY project in simulation S2 (NEE output) (**b**, **e**), and FLUXCOM models (**c**, **f**). Note that pixels where there is only a dry or only a wet season within a year are excluded. Triangles indicate that using simulated soil moisture by DGVMs or water availability index (WAI) for FLUXCOM models as terrestrial water storage instead of using observation-based terrestrial water storage would change the sign of C^TWS^.

Although the contribution of NEE in northern ecosystems to the global r_T_ plays a critical role in determining the dominant driver of the global NEE_IAV_, the magnitude of both the global r_TWS_ and r_T_ is mainly contributed by the tropical ecosystems (71–90% and 63–90%, respectively; Fig. 2b). Interannual variability of NEE in tropical ecosystems is much larger than that in the extra-tropics, and thus contributes more to the global NEE_IAV_^2–4^. Given that the impacts of climate variations on carbon dynamics in the tropics depend on hydrothermal conditions^8^, to gain more understanding of annual tropical C^TWS^ and C^T^, we separated these two contributions into dry and wet seasons (see Methods). Results from all three approaches show that, overall, tropical C^TWS^ has similar value between the dry and the wet seasons, and similarly, tropical C^T^ has similar value between the dry and the wet seasons (Supplementary Fig. 1). Moreover, in each tropical continent, albeit with uncertainties, C^TWS^ and C^T^ during the dry season are also similar to those during the wet season, respectively (Supplementary Fig. 2). Further, the spatial distributions of the absolute values of tropical C^TWS^ and C^T^ during the dry and wet seasons are strongly related to the magnitude of NEE_IAV_ (Fig. 5 and Supplementary Fig. 3). Regions with large absolute values of C^TWS^ and C^T^ shown in Fig. 5 also have large NEE_IAV_ in the same season. There is a concurrence of large C^TWS^ and strong interannual variations of NEE in savannas of southern Africa during the dry season (Fig. 5a–c and Supplementary Fig. 3). This region seems to be a hotspot of NEE_IAV_, and the large C^TWS^ and NEE_IAV_ during the dry season reflect a tight relationship between NEE and TWS at interannual timescales (Supplementary Fig. 4). More water availability during the dry season can induce a larger enhancement of vegetation productivity over ecosystem respiration, thus facilitating more net carbon uptake in this region^24^.

We investigated whether the general length of the dry season in tropical regions could partly explain the spatial and seasonal variations of NEE_IAV_ (Supplementary Figs. 3 and 5) and thus those of C^TWS^ and C^T^. Specifically, we found that regions with large absolute values of C^TWS^ and C^T^ in the dry season have longer dry seasons and similarly for the wet season (Fig. 5 and Supplementary Fig. 5). This finding indicates that future changes in the length of the dry season in tropical regions could affect the spatiotemporal patterns of C^TWS^ and C^T^ (ref. ^53^). In addition, we found negative values of C^TWS^ or C^T^ in some locations, e.g., for tropical Asia (Fig. 5), which result from the covariations between NEE_IAV_ in those regions and the IAVs of global TWS or global T (see Methods). Two mutually exclusive mechanisms could explain the negative values of C^TWS^ or C^T^ (see Methods). One is that years with more water availability or lower temperature do not enhance carbon sink in those regions. A second possibility is that local water availability or temperature variations are in the opposite direction of the global fluctuations (see Methods). The negative local C^TWS^ and C^T^ values imply a spatial compensation with positive values elsewhere in the tropics (Supplementary Note 2), modulating the C^TWS^ and C^T^ in the whole tropics.

## Discussion

**Fig. 6:**
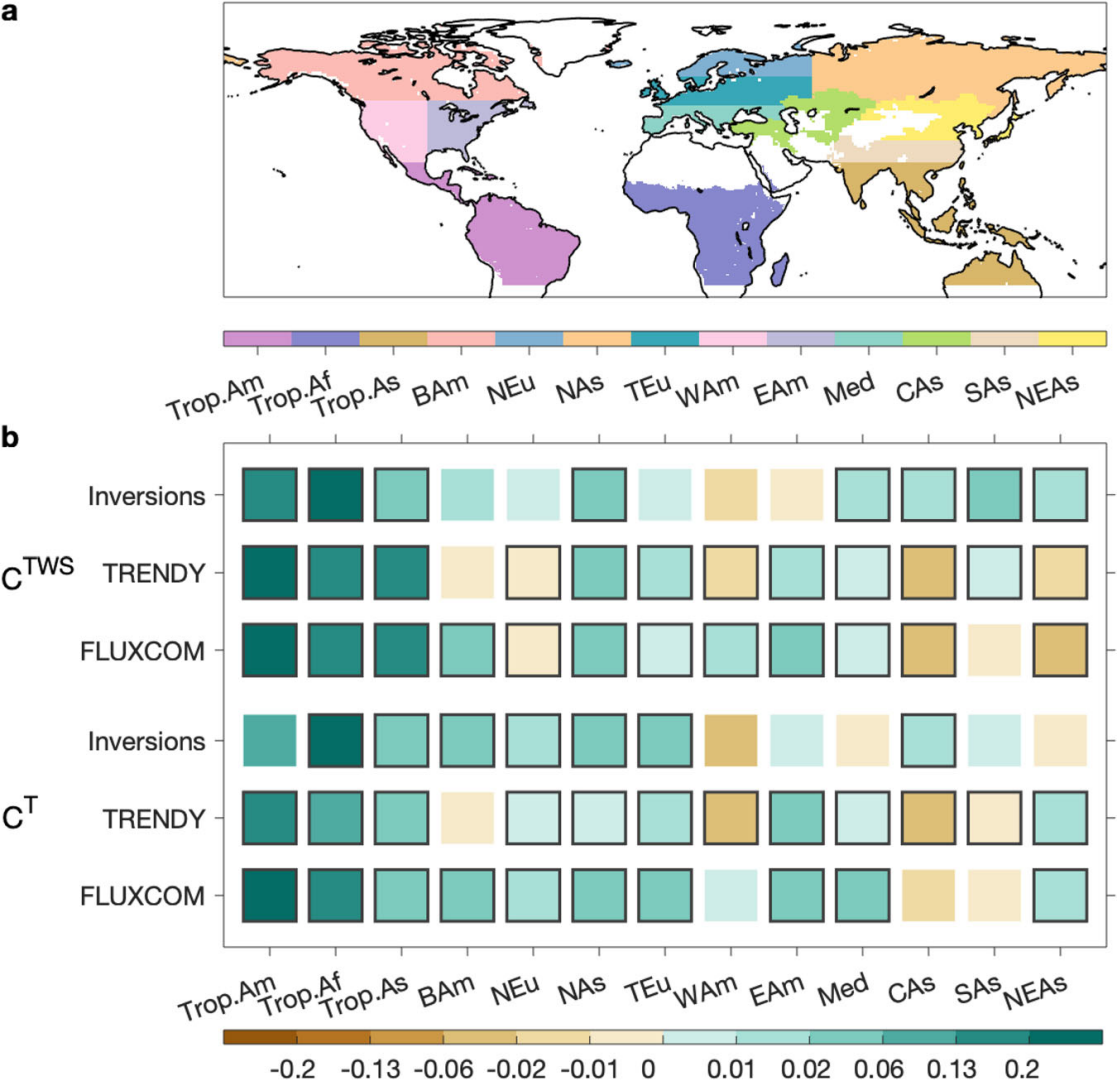
C^TWS^ and C^T^ in tropical continents and northern extra-tropical subregions. C^TWS^ and C^T^ indicate contributions to the global correlations of interannual variability of net ecosystem exchange against terrestrial water storage and temperature, respectively. As shown in panel **a**, the tropics is divided into three continents (Trop.Am tropical America, Trop.Af tropical Africa, Trop.As tropical Asia, with the latter including northern Australia). Northern Hemisphere (NH, >25°N) is divided into ten subregions. BAm Boreal America, NEu North Europe, NAs North Asia, TEu Temperate Europe, WAm Western North America, EAm Eastern North America, Med Mediterranean, CAs Central Asia, SAs South Asia and subtropical China, and NEAs Northern East Asia. For atmospheric inversions and FLUXCOM models, the black edges of the rectangles in panel **b** indicate that the signs of the C^TWS^ or C^T^ are the same among all models. For DGVMs from the TRENDY project, the black edges of the rectangles in panel **b** indicate that the signs of C^TWS^ or C^T^ derived from more than 10 out of 14 models are consistent with those from the model ensemble mean.

We have shown that the relative magnitude of C^TWS^ and C^T^ in the NH is critical to determine the dominant driver of the global NEE_IAV_ (Fig. 2a). However, there are considerable uncertainties in the estimate of C^TWS^ and C^T^ in subregions of NH (Supplementary Note 1). Specifically, we found little consensus between the three approaches in estimating C^TWS^ and C^T^ for many subregions in the NH (Fig. 6). Such regional discrepancies in annual C^TWS^ and C^T^ could partly result from seasonal compensation differences. For example, atmospheric inversions estimate larger C^TWS^ values than the other two approaches in Asian regions (i.e., North Asia, northern East Asia, Central Asia, South Asia, and subtropical China; Fig. 6), inducing a larger C^TWS^ in the NH (Fig. 2b). The large annual C^TWS^ in Asian regions, as estimated by atmospheric inversions, links to the positive values of spring C^TWS^ in these regions, which add to C^TWS^ in other seasons (Supplementary Fig. 6). By contrast, DGVMs from TRENDY project in simulation S2 and FLUXCOM models suggest the negative values of spring C^TWS^ in Asian regions, weakening the annual C^TWS^ (Supplementary Fig. 6). DGVMs from TRENDY project and FLUXCOM models suggest that increased water availability in spring can enhance the net land carbon uptake in East Asia and the arctic region of Asia (Fig. 4e, f; green and blue areas), by promoting the photosynthesis of vegetation^10,54^. Yet this is not supported by NEE values derived from atmospheric inversions (Fig. 4d; brown and pink areas).

In the tropics, discrepancies among the three approaches can be seen when analyzing continental contributions to the global r_TWS_ and r_T_ (Fig. 6 and Supplementary Fig. 2). Results from the three approaches show that both tropical Africa and tropical America generally contribute more to the global r_TWS_ and r_T_ than tropical Asia (including northern Australia, as shown in Fig. 6a). But large uncertainties remain in quantifying the continental contributions of tropical Africa and tropical America (Fig. 6 and Supplementary Fig. 2). Palmer et al.^17^, using inversions based on remotely-sensed column CO_2_ mole fraction retrievals, found some evidence of a substantial role of tropical Africa in the variability of the overall tropical NEE. However, that study was limited to a period of four years only. The longer NEE time series of atmospheric inversions based on in situ surface network used here show the large contributions of tropical Africa hold for the past four decades. In tropical Africa, fire is not likely to be the cause of net carbon flux anomalies in the recent few years^17,55^, implying that the control of NEE_IAV_ is the balance between photosynthesis and respiration. Besides the large contributions of tropical Africa, DGVMs and FLUXCOM models also estimate large C^TWS^ and C^T^ of tropical America (Fig. 6). This is probably because of the high NEE_IAV_ in tropical America estimated by DGVMs and FLUXCOM models^4,10^, reflecting the high sensitivity/vulnerability of tropical forests to climate anomalies^56^. The uncertainty in the contributions of three tropical continents may be largely from the spatial compensation effects (Supplementary Note 2).

To provide more accurate assessments of the spatial compensation for C^TWS^, or for C^T^, a key priority is reducing the uncertainties of the three approaches in estimating tropical NEE_IAV_. Atmospheric inversions assimilating surface measurements of atmospheric CO_2_ have potentially low capacity to separate the IAV signal between tropical continents, given the sparsity of stations recording atmospheric CO_2_ in the tropics^57^. The NEE estimate by the three atmospheric inversions is thus likely influenced by the prior settings in tropical regions. This can be partly remedied by assimilating total CO_2_ dry air mole fraction retrievals from satellites, as these have better coverage of the tropics^30,58^. For the FLUXCOM models, besides limitations from relatively few training samples in the tropics, the estimation of NEE is affected by both specific machine learning methods and in particular, the calculation of the water availability index (WAI) used as a predictor of NEE^33,46^. The soil water storage capacity in the bucket model used to simulate WAI in FLUXCOM is constant and relatively small^46^. By contrast, DGVMs calculate their own soil moisture, aiming to better capture the actual water availability and water stress on NEE. Uncertainties in simulating the NEE variability in DGVMs are thus mainly derived from the model structure, including soil hydrology and limitations of soil moisture and atmospheric dryness on plant photosynthesis and soil respiration^32,59,60^.

Atmospheric inversions and DGVMs from the TRENDY project indicate a dominant role of TWS on the global NEE_IAV_. However, TWS anomalies in some tropical regions relate to water storage in lakes, floodplains, and wetlands, which should have less effect on the carbon uptake than soil moisture^61^. This suggests a stronger relationship of NEE_IAV_ with simulated soil moisture in DGVMs or with WAI in FLUXCOM models than with ﻿observation-based TWS in tropical regions (Supplementary Figs. 7 and 8). This finding is also because these two model-based approaches are more consistent with their own derived soil moisture than with the independently observed TWS. Nevertheless, results from DGVMs and FLUXCOM models when considering their own simulated soil moisture, also support that the dry season length controls the absolute values of C^TWS^ in the tropics (Fig. 5 and Supplementary Fig. 9), although showing different spatial compensation patterns compared to results where C^TWS^ is calculated from observation-based TWS (Fig. 5; Supplementary Fig. 9; and Supplementary Note 2). In addition, even though the correlation between IAV of TWS reconstructed from GRACE satellite observations and IAV of the simulated soil moisture or water availability by the models is relatively weak in boreal and Arctic regions of the NH (Supplementary Fig. 10), it has little influence on the assessment of the dominant driver of NEE_IAV_ in the NH (Supplementary Fig. 7).

Our study revealed how the interannual NEE variations in different regions and seasons contribute to the overall apparent controls of TWS and T on the global NEE. At the global scale, both atmospheric inversions and DGVMs suggest that TWS exerts a stronger control on the NEE than T. This finding of a stronger TWS-dependence is supported by the tight relationship between interannual variations of the data-led global NEE estimated by atmospheric inversions and observed CGR variations^4^. The weaker T control primarily results from the seasonal compensation that the negative impacts of T on spring NEE offset the positive impacts on summer NEE over the northern ecosystems. However, a weaker spring-summer compensation of T effects in FLUXCOM models instead makes them produce a stronger T control than TWS on the global NEE. We note that the weak seasonal compensation is not supported by local flux tower measurements, which show a larger net carbon uptake by land in a warmer spring across northern ecosystems^16,43^. Nevertheless, weaker seasonal compensation effects over northern ecosystems may emerge in the future, due to the reduced chilling or growing light limitations in spring^44^ and to the accelerating carbon release from permafrost thawing or from more frequent summer heatwaves^38,62^. A weaker seasonal compensation effect in northern ecosystems could strengthen the T control in the global land carbon uptake variability. Hence monitoring how the seasonal compensation effects are changing in the Northern Hemisphere, supported by improved process modeling, will help better inform society about the emergence of any altered future carbon–climate feedbacks.

Atmospheric inversions, DGVMs from the TRENDY project, and FLUXCOM models all agree on the importance of the tropical contributions to the global NEE-TWS and NEE-T relationships. Here we show that this pan-tropical influence results from almost equal contributions from the dry and wet seasons. Our study highlights the role of dry season length in determining the spatial and seasonal pattern of the tropical contribution. To eventually quantify the contribution of tropical NEE during different seasons and at a finer spatial scale, a more accurate assessment of the spatial compensation of the contributions is needed. At present, the accuracy of tropical NEE estimates from atmospheric inversions and FLUXCOM models at fine resolution is limited by sparse atmospheric CO_2_ observations and eddy-covariance measurements in the tropics, respectively^33,57^. Assimilating satellite measurements of total CO_2_ dry air mole fraction into inversion algorithms has the potential to improve NEE estimates of atmospheric inversions at small spatial scales^30,58^. For DGVMs, the poor calibration or even lack of representation of key carbon cycle processes, such as nutrient limitations and access of roots to groundwater, could potentially induce biases in estimating NEE variations^37,63^. Given these different sources of uncertainty in simulating NEE among the three approaches, we emphasize the importance of comparing independent data sources and methods in determining regional carbon cycle attributes, as done in this study. In summary, we have shown that global annual variations in NEE may be more linked to fluctuations in terrestrial water storage than temperature, although this depends on a selected strand of evidence. Hence, we determined where data streams and model projections differ in estimating seasonal and regional NEE, and offsets in their variations. Our research provides guidance for future measurement campaigns and model development, which will lower further uncertainty on the dominant drivers of large-scale variations in NEE.

## Methods

### Atmospheric CO_2_ inversions

We used gridded net carbon fluxes for the period of 1979–2016 from two long-term atmospheric inversion models: CAMS version 17r1 (ref. ^28^) and Jena CarboScope version s76oc_v2020 (ref. ^29^). The two inversions both assimilated surface measurements of atmospheric CO_2_. The spatial resolutions of net carbon flux from CAMS and Jena CarboScope are 1.9°latitude × 3.75°longitude and 4°latitude × 5°longitude, respectively. We used the remapped monthly output with a spatial resolution of 1° × 1°. We also used another Jena CarboScope inversion called NEE-T-TWS (version sEXTocNEETTWS_79r18_v2020) for the period of 1979–2016. In the NEE-T-TWS inversion, a multilinear regression term related to T and TWS with inversely adjusted coefficients replaces the interannual NEE variations^16,31^. For some comparison, we used the CAMS inversion assimilating total column CO_2_ dry air mole fraction retrievals from the Japanese Greenhouse gases Observing SATellite (GOSAT) for the time period of 2010–2016 (ref. ^30^). For comparison with the inversion based on GOSAT retrievals, CAMS assimilating surface measurements is also limited to 2010–2016 (named Surf). We defined the net carbon flux as NEE and a positive value of NEE as a net carbon release from land.

### Dynamic global vegetation models

We used monthly net carbon fluxes estimated by 14 dynamic global vegetation models (DGVMs) from TRENDYv7 project^1,32^. The 14 models are CABLE-POP^64^, CLASS-CTEM^65^, CLM5.0^66^, DLEM^67^, ISAM^68^, JSBACH^69^, LPJ^70^, LPX-Bern^71^, OCN^72^, ORCHIDEE-CNP^73^, ORCHIDEE-Trunk^74^, SDGVM^75^, SURFEX^76^, and VISIT^77^, which have a monthly output covering the period of 1979–2016 (ref. ^1^). Nine of the 14 models include a fire module. The effects of carbon–nitrogen interactions are considered in nine models while few models include the phosphorus-cycle module such as ORCHIDEE-CNP. There is no model in TRENDYv7 specifically considering the tropical wetland hydrology. For permafrost, no TRENDYv7 DGVMs have explicitly considered it in the simulations. In addition, each model includes deforestation activities and afforestation activities or forest regrowth after cropland abandonment, as well as more generally land cover change. For a complete summary of the models, we refer to Le Quéré et al.^1^. Three simulations (S1, S2, and S3) were designed in the TRENDY protocol. DGVMs in S3 are forced by varying CO_2_, climate change, and land cover change while those in S2 are forced by varying CO_2_ and climate change. Differences of carbon fluxes between S2 and S3 are thus from the effects of land use change. We used the NEE outputs in simulation S2 of the TRENDY protocol and compared the results with the net biome productivity (NBP) output in simulation S3. All variables are remapped to a spatial resolution of 0.5° × 0.5°. We mainly used the IAV of modeled NEE in a comparison with that of the NEE from atmospheric inversions and FLUXCOM models.

### FLUXCOM global carbon flux dataset

We used data-driven NEE retrievals from the FLUXCOM model ensemble from an upscaling flux tower dataset using satellite and meteorological/climate forcing (RS+METEO)^10,33,46^. The meteorological forcing dataset used is CRU-NCEP v8 (ref. ^78^). FLUXCOM models use several products from the Moderate Resolution Imaging Spectroradiometer (MODIS), including MOD11A2 land surface temperature, the MOD13A2 vegetation index, MOD15A2 leaf area index and fraction of absorbed photosynthetic active radiation, and bidirectional reflectance distribution function-corrected surface reflectance of MCD43A2 and MCD43A4 (ref. ^46^), which can be obtained from http://daac.ornl.gov/MODIS/. The water availability index (WAI), which is calculated by a bucket model^33,46^, is also used in driving the machine learning algorithms. The bucket model considers a simple water budget without changes in snow cover, permafrost, and runoff^13,46^. FLUXCOM estimates gridded NEE based on satellite and meteorological/climate forcing data, using three machine learning methods (random forests, artificial neural networks, and multivariate adaptive regression splines) to upscale the NEE from measurements at eddy-covariance sites^46^. The machine learning is based on both the spatial and temporal gradients of observed NEE. More accurate predictions of NEE from FLUXCOM models can be obtained in regions with more eddy-covariance sites, such as temperate and boreal forests^46^. More observations in the tropical and arctic ecosystems are needed. We used the monthly NEE output of FLUXCOM models with a spatial resolution of 1° × 1° and covering the period of 1979–2016.

### Climate and terrestrial water storage data

We mainly used terrestrial water storage (TWS) as a proxy for water availability. The TWS dataset used in this study is reconstructed using a statistical model based on GRACE observations of TWS (GSFC dataset), and the relationship between climate drivers (MSWEP precipitation dataset and ERA5 air temperature dataset) and TWS^34^. Monthly reconstructed TWS covers the period of 1979–2016 with a spatial resolution of 1° × 1°. The main analyses in this study (Figs. 1–6) are based on this reconstructed TWS time series. For some comparisons, we also used the simulated soil moisture by DGVMs and the water availability index in FLUXCOM models derived from a soil water balance model forced by precipitation and evapotranspiration^10,46^. The correlation between TWS and simulated soil moisture or water availability index was estimated (Supplementary Fig. 10). Monthly gridded air temperature, precipitation, and potential evapotranspiration (PET) data for 1979–2016 are obtained from the Climatic Research Unit (CRU TS4.02), with a spatial resolution of 0.5° × 0.5° (ref. ^35^).

### Dry and wet seasons

The dry season in each grid cell is defined as the period when monthly PET is larger than precipitation in this pixel. The period in which monthly PET is lower than precipitation is defined as the wet season. Our analysis of the impacts of TWS and T on the tropical NEE_IAV_ during the dry and wet seasons excluded the pixels where there is only dry or only wet season within a year (Supplementary Fig. 5).

### CO_2_ growth rate (CGR)

We used atmospheric CO_2_ mole fraction data from the Greenhouse Gas Marine Boundary Layer Reference of the National Oceanic and Atmospheric Administration Earth System Research Laboratory (NOAA/ESRL)^79^. This dataset is constructed using measurements of weekly air samples from the Cooperative Air Sampling Network. We calculated the difference of CO_2_ mole fraction in January of this year and the following year to obtain the annual CGR.

### Correlation between global annual NEE and TWS or T (r_TWS_ or r_T_)

To obtain the IAV of TWS, T, and NEE, we removed the long-term trend using linear regression from the monthly time series and summed the values of each year into the yearly anomalies. Then the Pearson’s correlation coefficient between yearly NEE and yearly TWS (r_TWS_) or T (r_T_) was calculated. We excluded the years 1982, 1991, and 1992 from the time series of NEE, T, and TWS for each pixel because volcanic eruptions affected the radiation and the relationship between carbon uptake and water availability.

### Contributions of regional and seasonal NEE to the global r_TWS_ and r_T_

We first analyzed the contribution of monthly NEE in each grid cell to the global r_TWS_ and r_T_. We considered extensive quantities for NEE so we can split the global annual NEE anomaly (*X*_*G*_, unit: PgC yr^−1^) into *n* grid cells and 12 months covering the entire land surface and whole years with ${\sum }_{i{=}1}^{n}\mathop{\sum }_{m{=}1}^{12}{x}_{i,m}={{X}}_{G}$, where the *x*_*i,m*_ is the NEE anomaly (unit: PgC month^−1^) in grid cell *i* and month *m*. The correlation coefficient between *X*_*G*_ and global mean T anomaly (*T*_*G*_) can be decomposed as:

1 $$\begin{array}{c}{{{{\rm{corr}}}}}\left({X}_{G},{T}_{G}\right)=\tfrac{{{{{{\rm{cov}}}}}}\left({X}_{G},{T}_{G}\right)}{{{{\sigma }}_{X}}_{G}{\cdot} {{{\sigma }}_{T}}_{G}}=\mathop{\sum }\limits_{i=1}^{n}\mathop{\sum }\limits_{m=1}^{12}\tfrac{{{{{{\rm{cov}}}}}}\left({x}_{i,m},{T}_{G}\right)}{{{{\sigma }}_{X}}_{G}{\cdot} {{{\sigma }}_{T}}_{G}}\\ =\mathop{\sum }\limits_{i=1}^{n}\mathop{\sum }\limits_{m=1}^{12}\tfrac{{{{{{\rm{corr}}}}}}\left({x}_{i,m},{T}_{G}\right){{{\sigma }}_{x_{i,m}}}{\cdot} {{{\sigma }}_{T}}_{G}}{{{{\sigma }}_{X}}_{G}{\cdot} {{{\sigma }}_{T}}_{G}}=\mathop{\sum }\limits_{i=1}^{n}\mathop{\sum }\limits_{m=1}^{12}{{{{\rm{corr}}}}}\left({x}_{i,m},{T}_{G}\right){\cdot} \tfrac{{{{\sigma }}_{x}}_{i,m}}{{{{\sigma }}_{X}}_{G}}\end{array}$$

where cov(*X*_*G*_, *T*_*G*_) is the covariance between *X*_*G*_ and *T*_*G*_. The ${\sigma }_{{X}_{G}}$ and ${\sigma }_{{T}_{G}}$ are the standard deviation of *X*_*G*_ and *T*_*G*_, respectively. The global r_T_ is thus the sum of the correlations between *x*_*i,m*_ and *T*_*G*_ weighted by the ratio between the standard deviation of *x*_*i,m*_ and the standard deviation of *X*_*G*_ (Eq. (1)). The contribution of *x*_*i,m*_ to the global r_T_ can be expressed as:

2 $${C}_{i,m}^{T}={{{{{\rm{corr}}}}}}\left({x}_{i,m},{T}_{G}\right)\cdot \frac{{\sigma }_{{x}_{i,m}}}{{\sigma }_{{X}_{G}}}$$

which relates to the global correlation as:

3 $${{{{{\rm{corr}}}}}}\left({X}_{G},{T}_{G}\right)=\mathop{\sum }\limits_{i=1}^{n}\mathop{\sum }\limits_{m=1}^{12}{C}_{i,m}^{T}$$

The method of calculating the contribution of *x*_*i,m*_ to the global r_TWS_ is similar but with a negative sign because the global r_TWS_ is negative.

4 $${{{{{{\rm{C}}}}}}}_{i,m}^{{{{{{\rm{TWS}}}}}}}=-{{{{{\rm{corr}}}}}}\left({x}_{i,m},{{{{{{\rm{TWS}}}}}}}_{{{{{{\rm{G}}}}}}}\right)\cdot \frac{{\sigma }_{{x}_{i,m}}}{{\sigma }_{{X}_{G}}}$$

where TWS_G_ is the global mean TWS anomaly. The magnitudes of ${C}_{i,m}^{{{\mbox{TWS}}}}$ and ${C}_{i,m}^{T}$ are mainly affected by the ratio between the standard deviation of *x*_*i,m*_ and the standard deviation of *X*_*G*_, especially in the tropics. The magnitudes of the correlation between local NEE_IAV_ and local TWS or T have a weak influence on the spatiotemporal patterns of C^TWS^ or C^T^ in tropical ecosystems (Fig. 5, Supplementary Figs. 4 and 11). The sign of ${C}_{i,m}^{{{\mbox{TWS}}}}$ or ${C}_{i,m}^{T}$ is influenced by the correlation between the IAV of local NEE and global TWS or global T, which is probably linked to the control of local TWS or local T in the local NEE_IAV_, as well as the relationship between the IAVs of climate drivers at local and global scales.

The contribution of annual or seasonal NEE to the global r_TWS_ or r_T_ can be calculated by the direct sum of monthly C^TWS^ or C^T^. For tropical ecosystems, we examined C^TWS^ and C^T^ during the dry and wet seasons. For northern ecosystems, we considered four seasons: boreal spring (March–May), summer (June–August), autumn (September–November), and winter (December, January, and February). The contribution of regional or latitudinal NEE to the global r_TWS_ or r_T_ is calculated by the direct sum of C^TWS^ or C^T^ in each grid cell of the region or the latitudinal band.

### Sensitivity of NEE_IAV_ to TWS and T for different seasons

To investigate the sensitivity of NEE_IAV_ to TWS and T in different seasons, we first separated the monthly time series of NEE, TWS, and T into 12 groups, corresponding to the 12 months of a year. Each group included the values for the same month for all years. Then the linear trend of the time series in each group was removed to obtain anomalies of NEE, TWS, and T. Groups of months belonging to the same season were summed to become one group representing the season. The new group thus includes the values of the same season for all years. The sensitivity of NEE_IAV_ to TWS (*a*^TWS^) and T (*a*^*T*^) for a given season (*s*) and grid cell (*i*) was estimated by linear multiple regression:

5 $${{{{{{\rm{NEE}}}}}}}_{i,s}={a}_{i,s}^{{{{{{\rm{TWS}}}}}}}\cdot {{{{{{\rm{TWS}}}}}}}_{i,s}+{a}_{i,s}^{T}\cdot {T}_{i,s}+\varepsilon$$

where NEE_*i,s*_, TWS_*i,s*_, and *T*_*i,s*_ are vectors of NEE, TWS, and T in a given season (*s*) and grid cell (*i*) during 1979–2016, respectively and *ɛ* is a residual term. ${a}_{i,s}^{{{{{{\rm{TWS}}}}}}}$ and ${a}_{i,s}^{T}$ indicate the sensitivities of NEE_IAV_ to TWS and T for a given season (*s*) and grid cell (*i*), respectively.

## Supplementary information

supplementary information

## Data Availability

The net land carbon fluxes of CAMS atmospheric inversions are available at https://ads.atmosphere.copernicus.eu/cdsapp#!/dataset/cams-global-greenhouse-gas-inversion. The Jena CarboScope inversions results are available from the Jena CarboScope website http://www.BGC-Jena.mpg.de/CarboScope/. The simulations from TRENDY DGVMs are available at https://sites.exeter.ac.uk/trendy. The FLUXCOM ensemble of carbon fluxes is available at www.bgc-jena.mpg.de/geodb/projects/Data.php. Monthly gridded air temperature, precipitation, and potential evapotranspiration data from the Climatic Research Unit can be accessed at https://crudata.uea.ac.uk/cru/data/hrg/. The reconstructed TWS dataset based on GRACE observations is accessible at 10.6084/m9.figshare.7670849. The CO_2_ mole fraction data from the Greenhouse Gas Marine Boundary Layer Reference of the NOAA ESRL is available at https://gml.noaa.gov/ccgg/mbl/data.php.

## Code availability

All computer codes for the analysis of the data are available from the corresponding author on reasonable request.
