# Supplementary material for: Regional and seasonal partitioning of water and temperature controls on global land carbon uptake variability
Source: Nat Commun. 2022 Jun 16;13:3469. doi: 10.1038/s41467-022-31175-w (PMC9203577; doi:10.1038/s41467-022-31175-w)
Supplement: Supplementary file 1 — supplementary information [file 41467_2022_31175_MOESM1_ESM.pdf]

Supplementary Information for

Regional and seasonal partitioning of water and temperature controls on global land carbon uptake variability

Wang et al.

This file includes:

Supplementary Notes 1–2, Supplementary Figures 1–11, and Supplementary References

Supplementary Note 1. Estimating regional C^{TWS} and C^{T} in the Northern Hemisphere using three different methods

We estimated C^{TWS} and C^{T} for ten sub-regions of the Northern Hemisphere (NH) using atmospheric inversions, dynamic global vegetation models (DGVMs) from TRENDY project in simulation S2 and FLUXCOM models. We found that both C^{TWS} and C^{T} had strong spatial heterogeneity, and their spatial distributions vary between three methods. Results from atmospheric inversions suggest that high-latitude regions, i.e., boreal America, North Asia, North Europe and temperate Europe, contribute the most to the global r_T (Fig. 6). FLUXCOM models attribute a large contribution of both high-latitude regions and eastern North America to the global r_T (Fig. 6). In contrast, the ensemble of DGVMs estimates a small C^{T} for high-latitude regions, which explains why northern ecosystems are found to have a much smaller contribution to the global r_T in DGVMs compared with other two approaches (Fig. 2b). This is probably because state-of-the-art land carbon cycle models can not explicitly consider permafrost dynamics and nutrient limitation in their simulations[1–3]. In addition, atmospheric inversions and DGVMs of TRENDY project estimate larger C^{TWS} and C^{T} in North Asia than those in boreal America, while FLUXCOM models suggest that those two regions contribute almost equally to the global r_{TWS} and r_T (Fig. 6). The assessment of NEE in North Asia using FLUXCOM model can be limited by few eddy-covariance sites[4] and similarly, the atmospheric CO_2 measurement stations used by atmospheric inversions are not dense enough to separate the signals from small sub-regions in Asia[5].

In mid-latitude regions, uncertainties of NEE estimation using the three methods can be also found. DGVMs suggest that temperate Europe and eastern North America contribute similarly to both the global r_{TWS} and r_T, which is not supported by atmospheric inversions and FLUXCOM models (Fig. 6). Atmospheric inversions estimate a larger C^{T} in temperate Europe

than that in eastern North America while FLUXCOM models suggest C^T in these two regions are similar. Moreover, results from FLUXCOM models show that C^{TWS} in temperate Europe is much lower than that in eastern North America, which is opposite with result from atmospheric inversions. The C^{TWS} estimated by atmospheric inversions is negative in eastern North America, which is suggested to be positive by other two approaches (Fig. 6). We investigated the causes of this negative C^{TWS} from atmospheric inversions by analyzing the seasonal C^{TWS} for this region. We found that compared with other two approaches, atmospheric inversions suggest a large negative value of C^{TWS} in spring and positive C^{TWS} values in other three seasons which are much lower (Supplementary Fig. 6). This disagreement of seasonal C^{TWS} between atmospheric inversions and other two approaches may be further explained by different assessments of water sensitivity of NEE in eastern North America[6] (Fig. 4). Limitations of atmospheric inversions in estimating NEE at small scales have been noted in previous studies and this study[7]. This probably can explain the different results of atmospheric inversions with other methods in eastern North America and Central Asia (Fig. 6).

Given that there are large uncertainties of the three methods in estimating NEE for sub-regions of the NH, it should be noted that our conclusions in the NH of this study are mainly derived from findings at the broad latitudinal bands. In addition, despite the high spatial heterogeneity of C^{TWS} and C^T, the C^T estimated by FLUXCOM models is consistently larger than C^{TWS} in almost all northern sub-regions except western North America (Fig. 6). This indicate that the larger C^T than C^{TWS} in the NH suggested by FLUXCOM models is widespread and thus can be explained by the weak seasonal compensation of C^T at large scales (Fig. 3).

Supplementary Note 2. Spatial compensation of C^{TWS} and C^T in the tropics

When the values of C^{TWS} or C^T in some regions are negative, the spatial compensation can

occur. For example, the negative local C^{TWS} and C^{T} values in tropical Africa estimated by DGVMs and FLUXCOM models imply a spatial compensation with positive values elsewhere in this continent (Fig. 5), which could reduce the overall values of C^{TWS} and C^{T} in tropical Africa. By contrast, the results from atmospheric inversions show a weak spatial compensation in tropical Africa, which further explains why they estimate a large influence of this region on the global r_{TWS} and r_{T} (Figs. 5 and 6). In addition, the three approaches robustly show a higher C^{T} from tropical Africa than from tropical Asia (Fig. 6), due to spatial compensation of local C^{T} in tropical Asia (Fig. 5).

When using simulated soil moisture by DGVMs or the Water Availability Index (WAI) used by FLUXCOM models as a proxy of TWS, the spatial compensation of C^{TWS} can be different from that calculated using observation-based TWS in some regions (Fig. 5). We could find such differences by the values of C^{TWS} over the Amazon during the wet season in results from FLUXCOM models (Fig. 5f, Supplementary Fig. 9f). The differences reflect the uncertainties of simulating water availability in the tropics, but they do not affect the overall finding that the dry season length has a strong influence on the absolute values of C^{TWS} during the dry and wet seasons in the tropics.

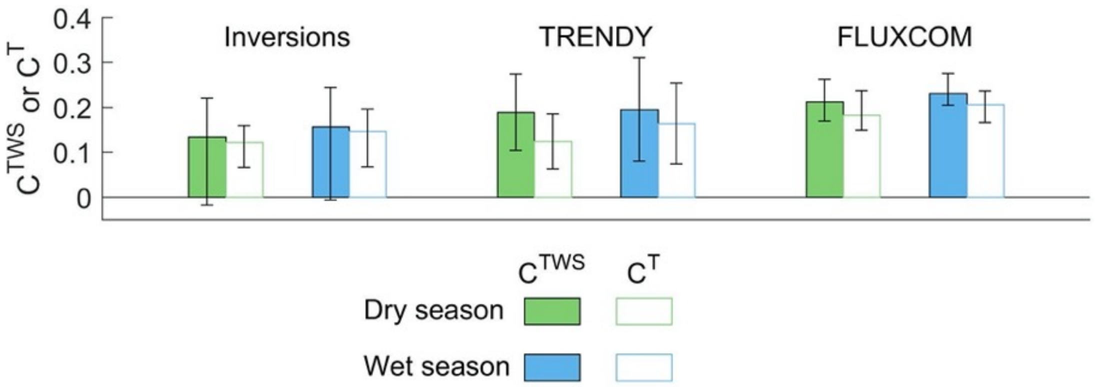

Supplementary Figure 1 Contribution of tropical NEE during the dry and wet seasons to the global r_{TWS} and r_T. Solid bars are the contribution to the global r_{TWS} (C^{TWS}) while open bars are the contribution to the global r_T (C^T). Green bars indicate the C^{TWS} or C^T during the dry season while blue bars indicate the C^{TWS} or C^T during the wet season. NEE is estimated by atmospheric inversions, DGVMs from TRENDY in simulation S2 (NEE output) and FLUXCOM models. For atmospheric inversions and FLUXCOM models, the error bars indicate the range of models while for DGVMs from TRENDY, the error bars indicate the 1-σ inter-model spread.

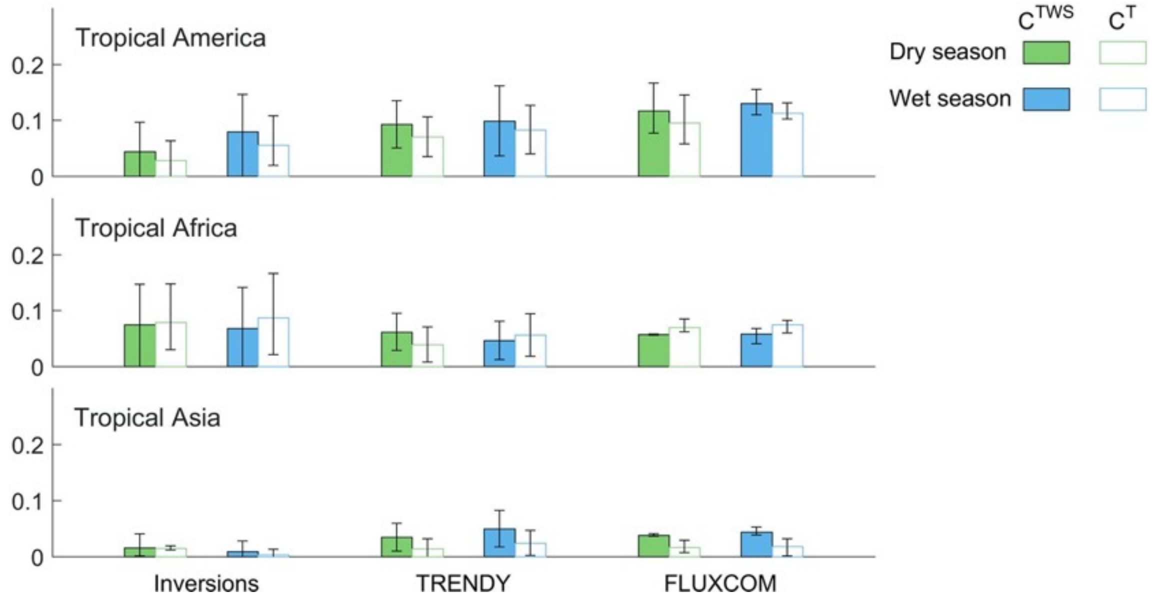

Supplementary Figure 2 Contribution of NEE in three tropical continents during the dry and wet seasons to the global r_{TWS} and r_T. Three tropical continents are tropical America, tropical Africa and tropical Asia, and the latter includes northern Australia. Solid bars are the contribution to the global r_{TWS} (C^{TWS}) while open bars are the contribution to the global r_T (C^T). Green bars indicate the C^{TWS} or C^T during the dry season while blue bars indicate the C^{TWS} or C^T during the wet season. NEE is estimated by atmospheric inversions, DGVMs from TRENDY in simulation S2 (NEE output) and FLUXCOM models. For atmospheric inversions and FLUXCOM models, the error bars indicate the range of models while for DGVMs from TRENDY, the error bars indicate the 1-σ inter-model spread.

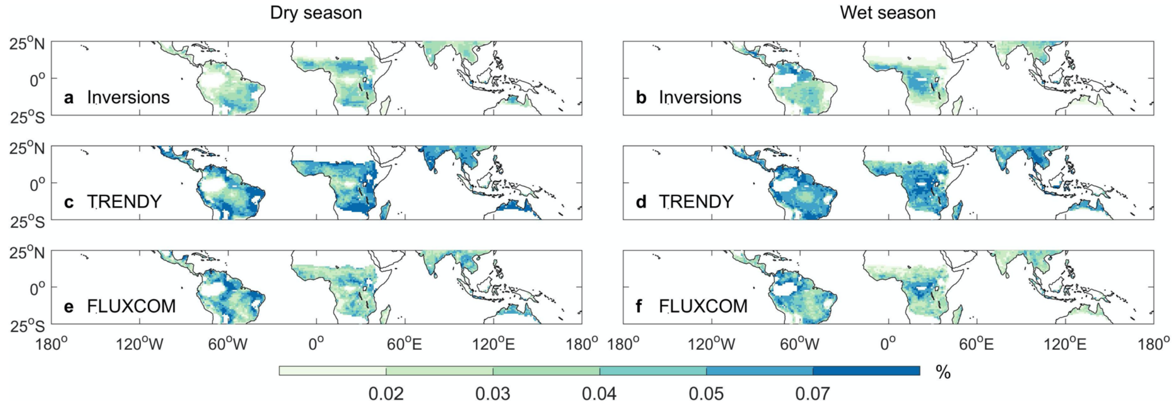

Supplementary Figure 3 Spatial distribution of the ratio between the standard deviation of NEE during the dry or wet season and the standard deviation of global annual NEE. NEE is estimated by atmospheric inversions (**a**, **b**), DGVMs from TRENDY in simulation S2 (NEE output) (**c**, **d**) and FLUXCOM models (**e**, **f**). Note that pixels where there is only dry or only wet season within a year are excluded.

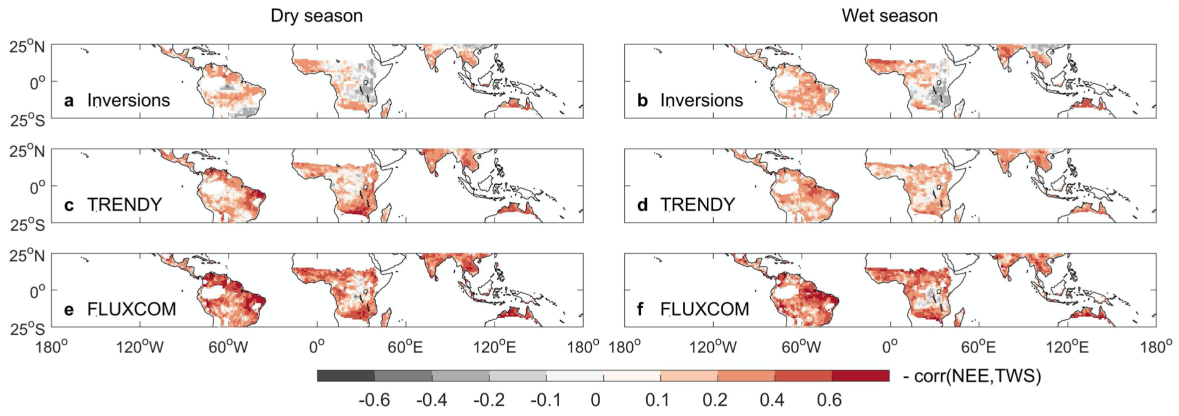

Supplementary Figure 4 Spatial distribution of correlation between NEE and TWS during the dry and wet seasons. NEE is estimated by atmospheric inversions (**a**, **b**), DGVMs from TRENDY in simulation S2 (NEE output) (**c**, **d**) and FLUXCOM models (**e**, **f**). The left column indicates the correlation of NEE to TWS during the dry season and the right column indicates the correlation to TWS during the wet season. Note that the correlation coefficient is shown with the opposite sign.

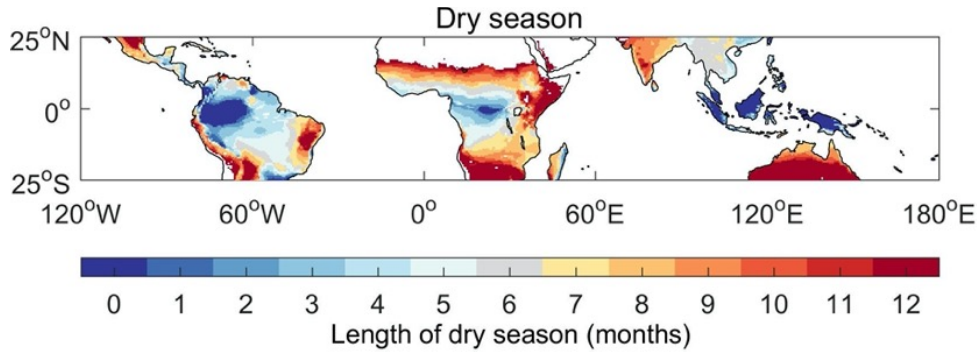

Supplementary Figure 5 Map in length of dry season for each pixel.

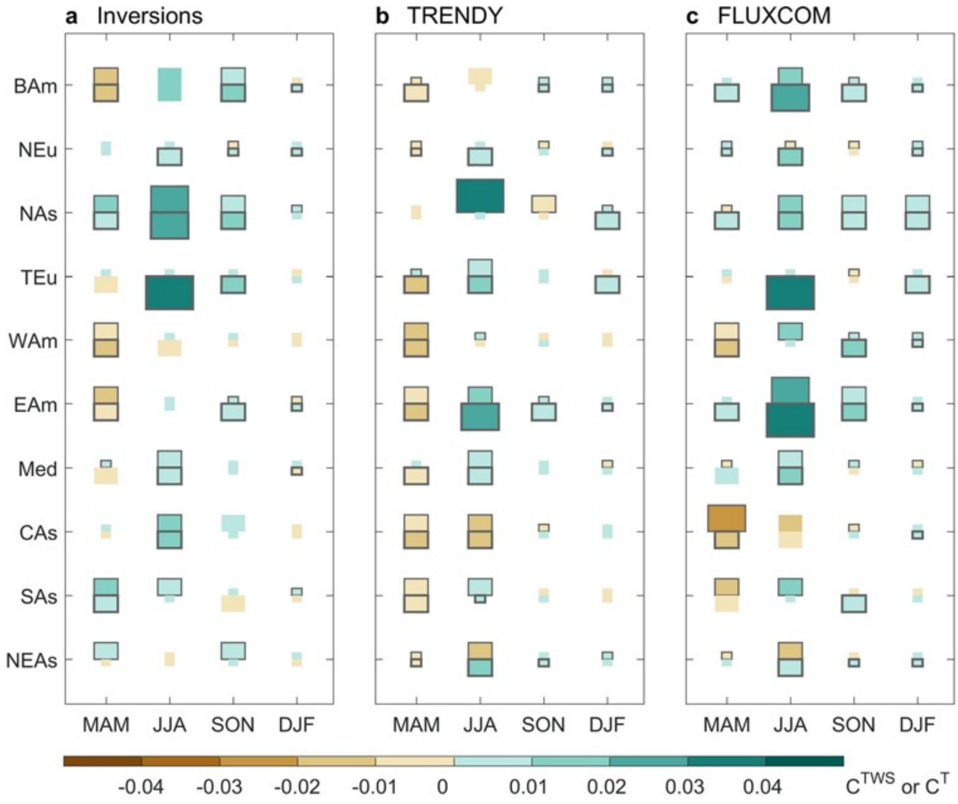

Supplementary Figure 6 C^{TWS} and C^{T} in northern extra-tropical sub-regions during four seasons. NEE is estimated by atmospheric inversions (**a**), DGVMs from TRENDY in simulation S2 (NEE output) (**b**), and FLUXCOM models (**c**). For each season and each region, two rectangles (C^{TWS} in the top and C^{T} in the bottom) are shown. The larger rectangles indicate the higher absolute values of the C^{TWS} or C^{T}. The four seasons are boreal spring (MAM, March–May), summer (JJA, June–August), autumn (SON, September–November), and winter (DJF, December and January–February). Northern Hemisphere (NH, >25°N) is divided into ten sub-regions (see map in Fig. 6). BAm: Boreal America, NEu: North Europe, NAs: North Asia, TEu: Temperate Europe, WAm: Western North America, EAm: Eastern North America, Med: Mediterranean, CAs: Central Asia, SAs: South Asia and subtropical China, and NEAs: Northern East Asia. For atmospheric inversions and FLUXCOM models, black edges of the rectangles indicate that the signs of the C^{TWS} or C^{T} are the same among all models. For DGVMs, black edges of the rectangles indicate that the signs of C^{TWS} or C^{T} derived from more than 10 out of 14 models are consistent with those from the model ensemble mean.

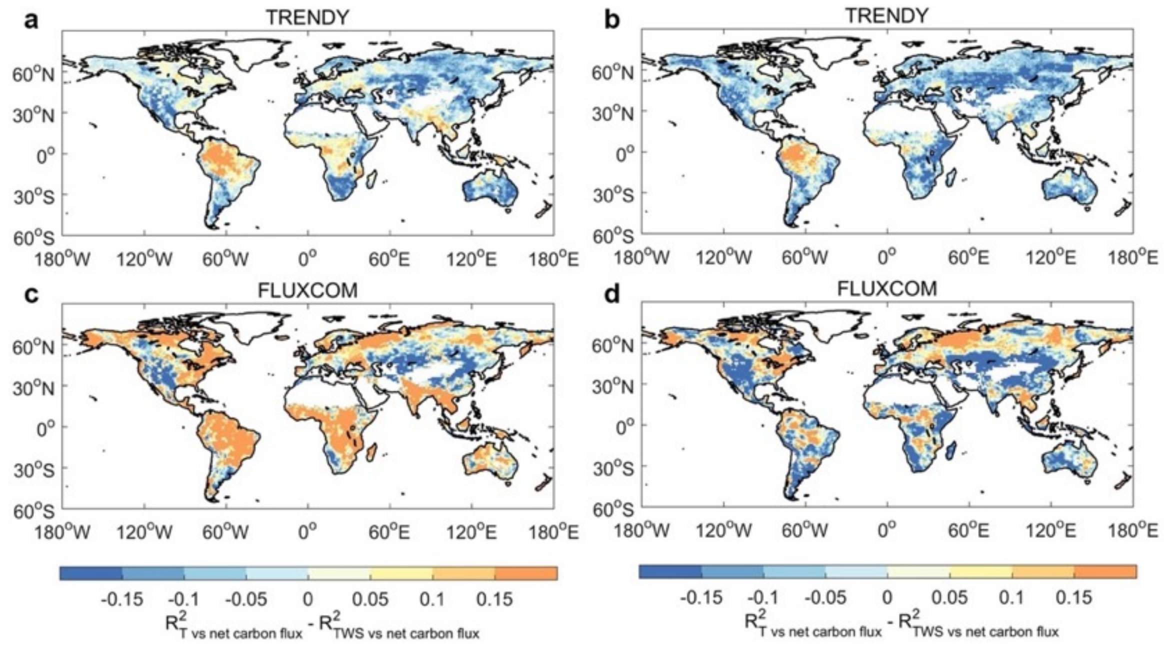

Supplementary Figure 7 Spatial distribution of difference between R^2 of NEE with T and that with water availability. Yellow pixels indicate NEE_{IAV} is more correlated to T than to water availability (represented by TWS) while blue pixels indicate more correlated to water availability. Note that in panels **a** and **c**, water availability is from GRACE TWS reconstruction and in panels **b** and **d**, water availability is simulated soil moisture by DGVMs from TRENDY and Water Availability Index (WAI) in FLUXCOM models, respectively.

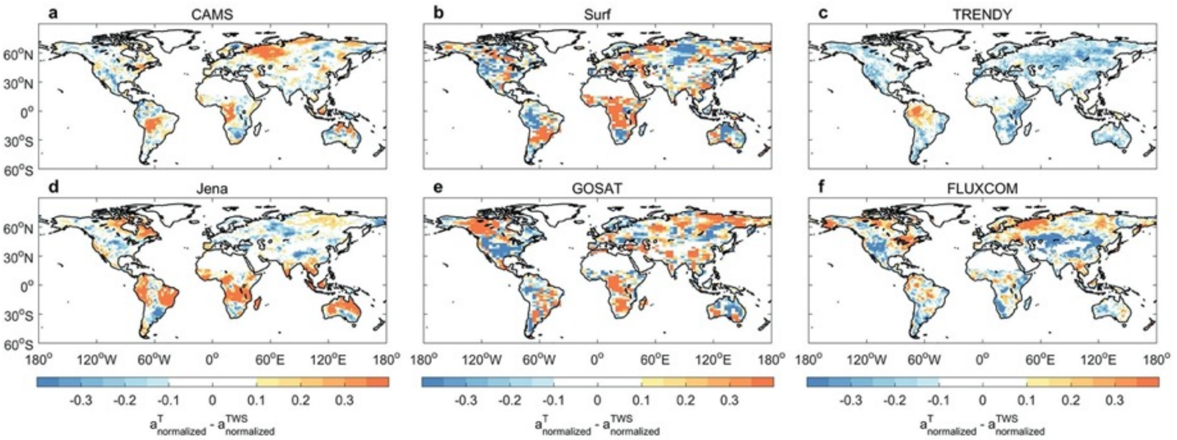

Supplementary Figure 8 Spatial distribution of difference between sensitivity of net carbon flux to T and that to water availability. The net carbon flux is estimated by atmospheric inversions (**a**, **b**, **d**, **e**), DGVMs from TRENDY project (**c**), and FLUXCOM models (**f**). Atmospheric inversions assimilate surface measurements (CAMS, Jena and Surf; see Methods) and total column CO_2 dry air mole fraction retrievals from the Japanese Greenhouse gases Observing SATellite (GOSAT). For comparison, the inversions based on surface observations (Surf) and GOSAT data have the same time span 2010–2016. Note that net carbon flux estimated by DGVMs from TRENDY (**c**) is the NBP output in simulation S3 (see Methods). Water availability is TWS reconstructed from GRACE observations when estimating the sensitivity of atmospheric inversed carbon flux to water availability. In the estimation of sensitivity of modeled carbon flux from DGVMs and FLUXCOM models, water availability is simulated soil moisture by DGVMs and Water Availability Index (WAI) in FLUXCOM models, respectively. Note that when calculating the sensitivities, all variables were normalized by the standard deviation, and then the absolute values of the sensitivities were used.

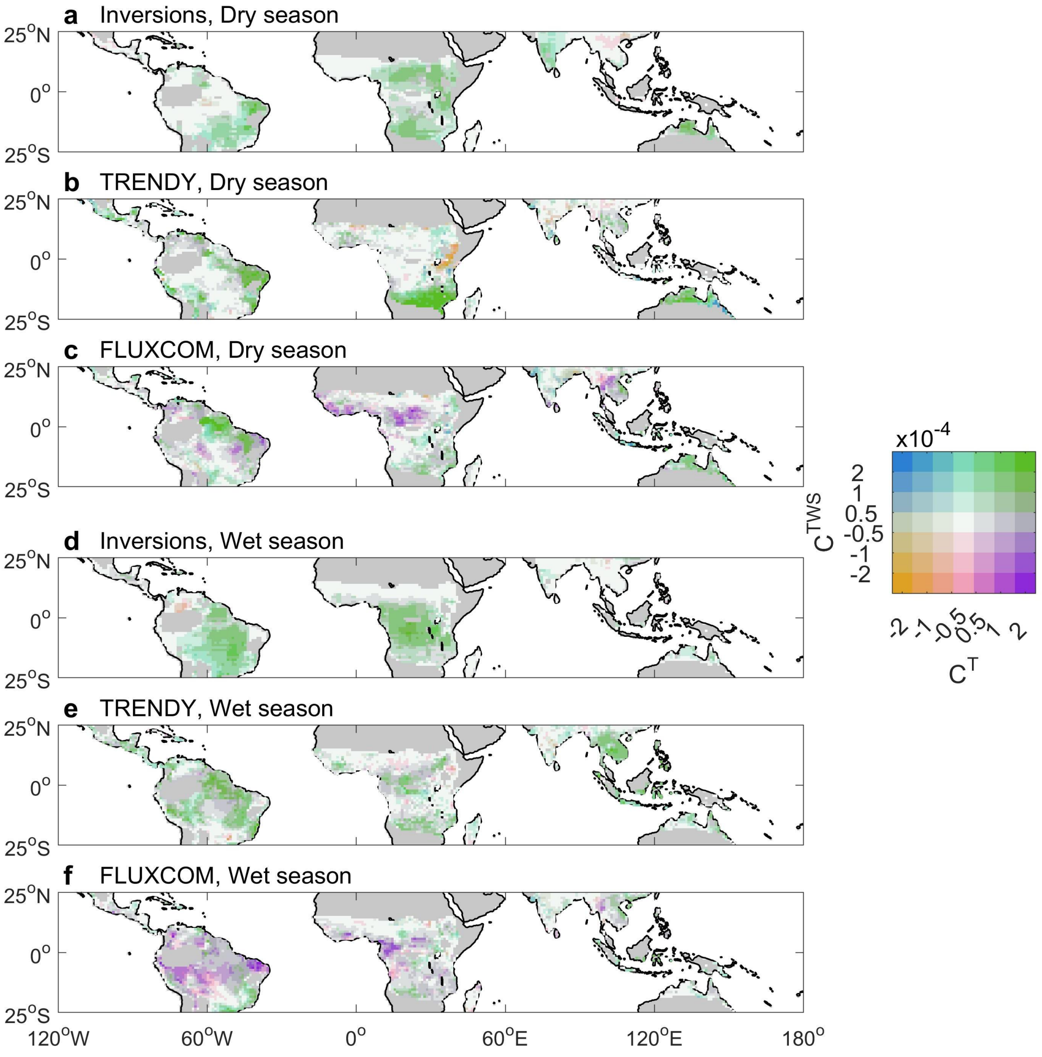

Supplementary Figure 9 Spatial distribution of the C^{TWS} and C^{T} in tropics during the dry and wet seasons. NEE is estimated by atmospheric inversions (**a**, **d**), DGVMs from TRENDY in simulation S2 (NEE output) (**b**, **e**), and FLUXCOM models (**c**, **f**). The pixels where there is only dry or only wet season within a year are excluded. Note that in panels **a** and **d**, TWS is reconstructed based on GRACE observations. By contrast, in panels **b** and **e**, simulated soil moisture in DGVMs from TRENDY is used as TWS while in panels **c** and **f**, water availability index (WAI) in FLUXCOM models is used.

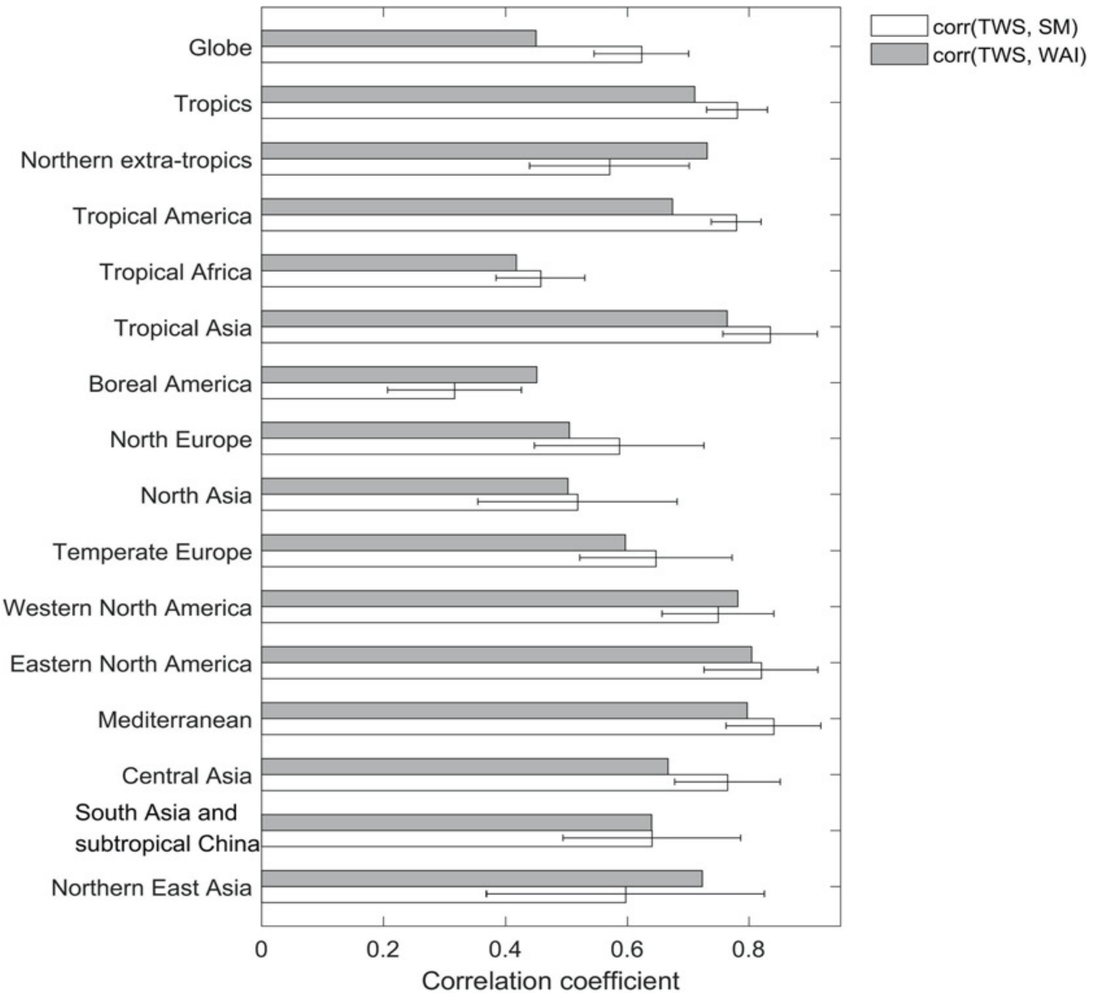

Supplementary Figure 10 Correlation coefficient between the reconstructed TWS based on GRACE observations and DGVMs-simulated soil moisture (SM) or Water Availability Index (WAI) used in FLUXCOM models. Error bars for correlation coefficient between reconstructed TWS and DGVMs-simulated soil moisture indicate the 1-σ inter-model spread.

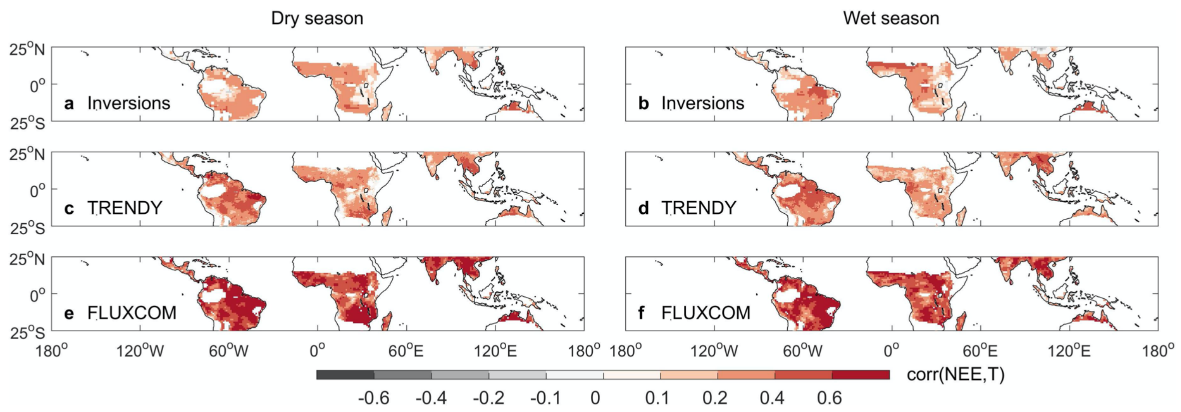

Supplementary Figure 11 Spatial distribution of correlation between NEE and T during the dry and wet seasons. NEE is estimated by atmospheric inversions (**a**, **b**), DGVMs from TRENDY in simulation S2 (NEE output) (**c**, **d**) and FLUXCOM models (**e**, **f**). The left column indicates the correlation of NEE with T during the dry season and the right column indicates the correlation with T during the wet season.

Supplementary References

1. Le Quéré, C. *et al.* Global Carbon Budget 2018. *Earth Syst. Sci. Data* **10**, 2141–2194 (2018).
2. Du, E. *et al.* Global patterns of terrestrial nitrogen and phosphorus limitation. *Nat. Geosci.* **13**, 221–226 (2020).
3. Turetsky, M. R. *et al.* Permafrost collapse is accelerating carbon release. *Nature* **569**, 32–34 (2019).
4. Jung, M. *et al.* Scaling carbon fluxes from eddy covariance sites to globe: Synthesis and evaluation of the FLUXCOM approach. *Biogeosciences* **17**, 1343–1365 (2020).
5. Peylin, P. *et al.* Global atmospheric carbon budget: Results from an ensemble of atmospheric CO_2 inversions. *Biogeosciences* **10**, 6699–6720 (2013).
6. Shiga, Y. P. *et al.* Forests dominate the interannual variability of the North American carbon sink. *Environ. Res. Lett.* **13**, 084015 (2018).
7. Bastos, A. *et al.* Impact of the 2015/2016 El Niño on the terrestrial carbon cycle constrained by bottom-up and top-down approaches. *Philos. Trans. R. Soc. B Biol. Sci.* **373**, 20170304 (2018).
